# Herbal medicines: a cross-sectional study to evaluate the prevalence and predictors of use among Jordanian adults

**DOI:** 10.1186/s40545-019-0200-3

**Published:** 2020-01-21

**Authors:** Faris El-Dahiyat, Mohamed Rashrash, Sawsan Abuhamdah, Rana Abu Farha, Zaheer-Ud-Din Babar

**Affiliations:** 1 College of Pharmacy, Al-Ain University, Alain campus, Al-Ain, P. O Box 64141, United Arab Emirates; 2 Department of Pharmaceutical and Administrative Sciences, School of Pharmacy, University of Charleston, Charleston, WV USA; 3 College of Pharmacy, Al-Ain University, Abu Dhabi campus, Al-Ain, United Arab Emirates; 4 Department of Biopharmaceutics and Clinical Pharmacy, Faculty of Pharmacy, The University of Jordan, Amman, Jordan; 5 Department of Clinical Pharmacy and Therapeutics, Faculty of Pharmacy, Applied Science Private University, Amman, Jordan; 6 Department of Pharmacy, University of Huddersfield, Huddersfield, UK

**Keywords:** Herbal medicines, Jordan, Attitude, Prevalence, Risk factors

## Abstract

**Introduction:**

Understanding why adults resort to herbal medicine can help in planning interventions aimed at increasing awareness regarding herbal use. This study sought to investigate the prevalence and to determine factors for predicting the use of herbal medicine among Jordanian adults.

**Methods:**

A cross-sectional study was conducted involving 378 older adults who were randomly selected from two different areas of Jordan. A questionnaire was used to gather data and validation criteria for validity and reliability of the content were tested by content and face validity in a panel of experts.

**Results:**

From a total of 500 invited participants, 378 completed the questionnaire. The prevalence of the use of of herbal products in this study was high at 80.2%. Herbal medicines use was not associated with any demographic factors other than age (*p* < 0.05). Moreover, the only associated health-related characteristic was the patient’s disease state including, notably, hypertension (*p* < 0.05). Reasons for not using herbal medicines as reported by nonusers included mainly a lack of belief in their efficacy (52.2%). Another two important reasons were that the individuals believed themselves to healthy and have no need for their use (31.3%) and the unavailability of enough information about the herbal medicines (29.7%). Finally, the most common side effects as reported by patients in this study were nausea and vomiting (9.3%), and, to a lesser extent, skin rash (2.1%).

**Conclusion:**

There is a high rate of use of herbal medicines in Jordan, especially among hypertensive patients. Therefore, there is a need to establish effective herbal medicine policies and health education programs to discuss the benefits and risks of herbal medicine use, with the aim of maximizing patient-desired therapeutic outcomes.

## Introduction

Herbal medicines are substances one can eat or drink and may be vitamins, minerals, or herbs or parts of these substances. They can be defined as ‘plants or plant parts used for their scent, flavour, or therapeutic properties’ [1]. Herbal medicines are distinct from drugs wherein they are exempted from needing to meet premarketing safety and efficacy standards required for conventional drugs to adhere to [2]. The use of herbal medicines has increased remarkably throughout the world, with many people now using these products for the treatment of many health problems in health care practice across different countries [3].

People report using herbal medicine to meet a variety of health care needs, including disease prevention and to cure chronic illnesses such as dyslipidemia, hypertension, diabetes, cancer, and inflammatory bowel diseases [4, 5].

The usage of herbal medicines in the world varies depending on location and the prevalence has increased recently. In the Arab world, similar rates have been found. About 80% of the population in Arab societies relies on herbal medicines for the prevention and treatment of illness [6]. For instance, in Egypt, 37% of the population reported using herbal medicines [7], while, in Saudi Arabia, a higher proportion of the population (73%) have used herbal medicines [8]. In Jordan, herbal medicine has maintained popularity as a result of historical, cultural, and psychosocial factors [9]. The most common reasons for using traditional herbal medicine are that it is inexpensive, more closely corresponds with the patient’s beliefs, avoids concerns about the adverse effects of chemical (synthetic) medicines, satisfies a need for more personalised health care, and allows for a greater public approach to health information [10].

It is hypothesised that as the use of herbal medicine increases among Jordanian adult populations so too do the occurrence of adverse effects and herbal drug interactions. Knowledge of the predictors of herbal use may help health care providers to identify patients at high risk who would be candidates for receiving additional guidance on the safe use of herbal medicines [11]. Such could further provide pathways for facilitating positive social changes by developing stricter governmental policies to ensure consumer safety and promote high-quality products and by driving the development of public awareness interventions about herbal use and related health risks.

The present study aimed to examine the prevalence and to identify factors predicting the use of herbal medicine among adults in Jordan. Understanding why adults resort to herbal medicine can help with planning interventions to increase awareness about herbal use. Such could also shed light on the importance of setting frameworks to regulate the entry into, distribution, and use of herbal medicine in the Jordanian market.

## Methods

### Study design, subjects, and setting

This was a cross-sectional study that was carried out in Jordan. Data collection period was from 10 March to 19 April 2017. During the study period, 500 Jordanian individuals were invited to participate in this study and to fill out an anonymous questionnaire designed to evaluate the nature of their herbal medicine use and to identify factors predicting their use of herbal medicine. Participants were Universities students and their family members. Universities staff and their family. The students were approached while participating in different classes. The study objectives were explained to them and they were informed that the study was to assess the knowledge and beliefs about the use of herbal medicine in Jordan.

### Questionnaire deployment and data collection

Data collection was carried out using self-administered questionnaires that were developed by the researchers based on questions extracted from previous studies [12, 13].

Content validity and face validity of the items questionnaire was evaluated in a panel of experts. Qualitative face validity was evaluated by asking the opinion of experts including a sample of the target group and 5 faculty members, assessed the questionnaire for appropriateness, complexity, attractiveness and relevance for the items. The items were edited and reworded based on their statements. Content validity was also evaluated by qualitative and quantitative methods. In the qualitative phase, we invited two expert panel to evaluate and discuss the essentiality of the questionnaire items, its wording and scaling, and its relevance. In quantitative method, content validity ratio (CVR) and content validity index (CVI) were tested for each item. If CVR was greater than the criterion of the Lawshe’s table [14] for each item, the item was weighed as essential; if not, it was omitted. According to the Lawshe table [14], an acceptable CVR value for 5 experts is 0.99.

The questionnaire was divided into four sections. The first section dealt with respondents’ acquisition, recommendations, and trust of currently available information on herbal medicines. The second part inquired about respondents’ attitudes towards herbal medicines The third part requested the health-related characteristics of study participants. The final section characterised the respondents’ demographics. The methods for response were organised differently, including using single-answer, multiple-answer (participants were allowed to choose more than one answer), and four-point Likert scale (i.e., 1 = strongly disagree, 2 = disagree, 3 = agree, and 4 = strongly agree) schemes.

### Ethical considerations

This study was conducted following the guidelines outlined in the World Medical Association’s Declaration of Helsinki [15]. Ethical approval for conducting this study was obtained from the Institutional Review Board Committee at Applied Science Private University.

The participation of members of the Jordanian public was strictly voluntary. Informed consent of the participants was obtained prior to study inclusion and no personal data of the participants are reported. The anonymity of respondents was preserved in the study, as the names of participants were not included.

### Sample-size calculation and sampling technique

A sample size calculation was performed using the following formula:

$$ \mathrm{n}=\mathrm{P}\times \left(100-\mathrm{P}\right)\times {\mathrm{z}}^2/{\mathrm{d}}^2 $$

Where P is the anticipated prevalence of students’ knowledge, d is the desired precision, and z is the appropriate value from the normal distribution for the desired confidence.

Using a 95% confidence level (CI), 10% precision level, and 50% anticipated prevalence of inappropriate knowledge, a minimum sample size of 96 people was considered as accurately representative for the purpose of this study. In this study, we tried to approach 500 subjects to increase the generalizability of the study. A convenience sampling technique was employed to approach students based on their accessibility and proximity to the researcher.

### Statistical analysis

All data were entered and analysed using SPSS© version 22 (IBM Corp., Armonk, NY, USA). Categorical variables were expressed as frequencies and percentages, while continuous variables were presented as means ± standard deviations (SDs). The chi-squared test was used to evaluate demographic and health-related characteristics associated with herbal medicines.

Multiple logistic regression analysis was used to identify attitude-related factors that best predicted the use of herbal medicines in the study population, using odds ratio (OR) values as a measure of association. A *p*-value of less than 0.05 was considered to be statistically significant.

## Results

The first draft of the questionnaire was formed through a grounded theory study and extensive literature review. The questionnaire was divided into four sections. The first section dealt with respondents’ acquisition, recommendations, and trust of currently available information on herbal medicines. The second part inquired about respondents’ attitudes towards herbal medicines. The third part requested the health-related characteristics of study participants. The final section characterised the respondents’ demographics.

In qualitative face validity, by consideration of the expert panel, four items were deleted due to content overlap. One item was also omitted due to complexity. In qualitative content validity, we changed two items according to the experts’ recommendations. In the quantitative stage, CVR of all the items was between 0.99, except for 4-items that had a CVR < 0.62 and therefore were deleted.

The CVI for each item scale was the proportion of experts who rated an item as 3 or 4 on a 4-point scale [16]. Clarity, simplicity, and relevancy of each item were scored in a four-point Likert scale (from 1: not relevant, not simple, and not clear to 4: very relevant, very simple, and very clear). Items with scores less than 0.7 were omitted. CVI of other items were between 0.8–1.

Construct validity of this questionnaire was evaluated by 378 respondents with mean age of 26.7 ± 5.60 years. Detailed demographic data of the study participants are as shown in Table 1. A total of 378 respondents responded to the questionnaire and the majority of them reported using herbal medicine (80.8%). The main reason for the nonparticipation of the remaining students (*n* = 122) was a lack of interest in the subject of the study. About two-thirds of respondents were female (69.6%). The majority had either bachelor or college degrees of education (62.9%) and had an annual income of less than 1000 (68.3%).

**Table 1:** Characteristics of study participants, the association between the use of herbal medicines and respondents’ characteristics (*n* = 378)

Characteristic	Total N(%)	Used Herbal Products n(%)	Chi square *P*-value
Gender			
Male	115 (30.4)	87 (85.6)	> 0.05
Female	263 (69.6)	216 (82.1)	
Age*			
15–25	187 (49.5)	148 (79.6)	< 0.05
26–35	69 (18.3)	49 (72.1)	
36–45	54 (14.3)	47 (87.0)	
46–55	44 (11.6)	41 (93.1)	
> 55	23 (6.1)	18 (78.3)	
Marital Status			
Single	200(52.9)	157 (79.3)	> 0.05
Married	169 (44.7)	139 (82.2)	
Divorced	8 (2.1)	6 (75.0)	
Education			
Uneducated	27 (7.1)	25 (92.6)	> 0.05
Post Graduate	45 (11.9)	34 (75.6)	
Bachelor Degree	157 (41.5)	126 (80.8)	
College	81 (21.4)	62 (76.5)	
High school	65 (17.2)	54 (84.4)	
Occupation			
Unemployed	52 (13.8)	38 (74.5)	> 0.05
Professional	104 (27.5)	84 (81.6)	
Student	134 (35.4)	105 (78.4)	
Housewife	53 (14.0)	46 (86.8)	
Retired	29 (7.7)	25 (86.2)	
Residence			
Amman	147 (38.9)	117 (79.6)	> 0.05
Zarqaa	158 (41.8)	129 (82.7)	
Others	73 (19.3)	56 (86.7)	
Income			
500 JD#	178 (47.1)	142 (80.7)	> 0.05
500–1000 JD	148 (39.2)	123 (83.1)	
> 1000 JD	30 (7.9)	24 (80.0)	

#1 JD = 1.4 USD

*P-value is ≤ 0.05

Table 2 shows responses pertaining to health-related characteristics and the use of herbal medicines. More than three-quarters of the study sample admitted using herbal medicines. The majority of participants rated their health as either excellent or very good (71.4%) but no significant association between the provided health rating and the usage of herbal medicines was observed. About 80% of the study population did not report the presence of any chronic disease, and there was no association between the presence of chronic illness and the use of herbal medicine found. The most prevalent chronic diseases among the study subjects were hypertension followed by diabetes (9.5 and 5.6%, respectively), and there was a statistically significant association between the type of chronic illness and the admitted use of herbal medicines. More than half of the respondents were somewhat unfamiliar with herbal medicines (52.6%). Among those who used herbs, about one-third were using them only during certain seasons, and approximately half of them reported used herbal remedies followed by vitamins and minerals, respectively (48.9 and 21.7%). The main reasons for using the products were to treat disease and maintain health (44.8%). Approximately 22% of consumers experienced side effects from using herbal medicines including, most commonly, vomiting and nausea (9.3%).

**Table 2:** Health-related characteristics of study participants (*N* = 378), Association between the use of herbal and respondents’ characteristics

Characteristic	N (%)	Users n(%)	Chi Square *p* value
Personal Health			> 0.05
Excellent	116 (30.7)	91 (78.4)	
Very good	154 (40.7)	128 (83.7)	
Good	98 (25.9)	76 (78.4)	
Fair	7 (1.9)	6 (85.7)	
Poor	3 (0.8)	2 (66.7)	
Have a Chronic Disease			
Yes	65 (17.2)	55 (84.6)	> 0.05
No	303 (80.2)	241 (80.1)	
Chronic Diseases Type*			
Hypertension	36 (9.5)	32 (88.9)	< 0.05
Diabetes	21 (5.6)	16 (76.2)	
Heart diseases	8 (2.1)	6 (75.0)	
Others	22 (5.8)	22 (100.0)	
Familiarity with the natural health products			
Not at all familiar	61 (16.1)		> 0.05
Somewhat unfamiliar	199 (52.6)		
Very familiar	101 (26.7)		
Used a Herbal products			
Yes	303 (80.2)		> 0.05
No	73 (19.3)		
Description of Use of frequency of using Herbal products			
Daily	50 (13.2)		> 0.05
Weekly	69 (18.3)		
Monthly	67 (17.7)		
Only during a certain season	130 (34.4)		
Type of herbal product used			
Vitamins/Minerals	82 (21.7)		> 0.05
Herbal remedies	185 (48.9)		
Homeopathic medicines	8 (2.1)		
Traditional medicines	33 (8.7)		
Probiotics	1 (0.3)		
Amino acids and essential fatty acids	4 (1.1)		
Use Of Herbals Reason			
Maintain Health	68 (18.0)		> 0.05
prevent Illness	49 (13.0)		
Treat Disease	101 (26.7)		
Supplement	37 (9.8)		
Others	62 (16.4)		
Experience Side Effect			
Yes	81 (21.4)		> 0.05
No	236 (62.4)		
Type of Side Effect			
Nausea	15 (4.0)		> 0.05
Vomiting	20 (5.3)		
Diarrhea	9 (2.4)		
Constipation	5 (1.3)		
Nervousness/Anxiety	10 (2.6)		
Dizziness	7 (1.9)		
Skin rash	8 (2.1)		
Others	5 (1.3)		

*P-value is ≤ 0.05

Table 3 indicates that the majority of consumers obtained herbal medicines from herbalists followed by from a pharmacy (37.8 and 23.0%, respectively). Herbal medicine use was mainly recommended by family and friends (39.7%) followed by pharmacists (17.7%) and mass media (12.4%). Pharmacists and medical doctors were the individuals most trusted to provide accurate information on herbal medicines (24.6 and 23.3%, correspondingly).

**Table 3:** Respondents’ responses regarding the acquisition, recommendations, and trust of currently available information on herbal products (*n* = 378)

Questions	n(%)	Chi square p value
Obtain herbal Products Pharmacy	87 (23.0)	> 0.05
Herbalist	143 (37.8)	
Health products store	6 (1.6)	
Supermarket	30 (7.9)	
Family member/Friend	44 (11.6)	
Others	7 (1.9)	
Who recommended herbal products^*^		< 0.05
Family member/Friend	150 (39.7)	
Medical Doctor	37 (9.8)	
Pharmacist	67 (17.7)	
Nurse	4 (1.1)	
Dietician	4 (1.1)	
Mass Media (TV/Radio/Newspaper)	47 (12.4)	
Others	8 (2.1)	
Trust to provide accurate information^*^		< 0.05
Medical Doctor	88 (23.3)	
Pharmacist	93 (24.6)	
Herbalist	59 (15.6)	
Nurse	1(.3)	
Dietician	10 (2.6)	
The natural health product manufacturer	5 (1.3)	
Family member/Friend	49 (13.0)	
Health products store	3(.8)	
Others	8 (2.1)	

*P-value is ≤ 0.05

Reported attitudes towards herbal medicines, as presented in Table 4, revealed that the majority of respondents agreed with six statements and disagreed with two statements. The reported disagreements were with the statements *if a herbal medicines is for sale to the public, I am confident that it is safe* and *herbal medicines are better for me than conventional medicines.* The strongest agreement was with the statement *herbal medicines can maintain and promote health* followed by that the respondents desired to know more about the safety and efficacy of herbal medicines and about the possibility of the use of herbal medicines to treat illnesses (83.3, 79.6, and 77.8%, respectively).

**Table 4:** Respondents’ attitudes towards Herbal Medicines (HM) (*n* = 372)

Statement	Agree	Disagree
HMs maintain and promote health	315 (83.3)	57 (15.1)
HMs can be used to treat illness	294 (77.8)	78 (20.6)
HMs are safe because they are made from natural ingredients	261 (69.0)	104 (27.5)
If a HM is for sale to the public, I am confident that it is safe	177 (46.8)	192 (50.8)
HMs are better for me than conventional medicines	181 (47.9)	186 (49.2)
A lot of the health claims made by the manufacturers of HMs are unproven	218 (57.7)	153 (40.5)
Do you agree that currently there is a lack of resources available to you regarding the use of herbal medicines	225 (59.5)	146 (38.6)
Would you like to know more about the safety and efficacy of herbal medicines	301 (79.6)	71 (18.8)

*HM* Herbal medicine

Multivariate logistic regression analysis outcomes comparing who agreed and disagreed about certain statements regarding herbal medicine use are shown in Table 5. The highest odds were found among people who agreed about the use of herbs to maintain health (OR: 3.9, 95% CI: 0.12–0.57), while the least significant odds were found among those who agreed with the statement *a lot of the health claims made by the manufacturers of herbal medicines are unproven* (OR: 0.515, 95% CI: 1.05–3.60). Other significant predictors were herbal medicines *can be used to treat illness* and *if a herbal medicineis for sale to the public*, *I am confident that it is safe* (*p* < 0.05).

**Table 5:** Logistic regression analysis of respondents’ attitudes towards Herbal Medicines (HM) predicting the difference between who agreed and disagreed about certain statements representing their attitudes (*n* = 372)

Statement	Agree	Disagree	P value	OR	95% C. I for EXP(B)
HMs maintain and promote health	315 (83.3)	57 (15.1)	0.001	3.90	(0.12–0.57)
HMs can be used to treat illness	294 (77.8)	78 (20.6)	0.049	1.98	(0.26–0.99)
HM products are safe because they are made from natural ingredients	261 (69.0)	104 (27.5)	0.079	1.78	(0.29–1.07)
If an HM is for sale to the public, I am confident that it is safe	177 (46.8)	192 (50.8)	0.041	0.519	(1.03–3.62)
HMs are better for me than conventional medicines	181 (47.9)	186 (49.2)	0.358	0.764	(0.74–2.33)
A lot of the health claims made by the manufacturers of HM products are unproven	218 (57.7)	153 (40.5)	0.036	0.515	(1.05–3.60)
Do you agree that currently there is a lack of resources available to you regarding the use of herbal medicines	225 (59.5)	146 (38.6)	0.516	0.813	(0.66–2.29)
Would you like to know more about the safety and efficacy of herbal medicines	301 (79.6)	71 (18.8)	0.260	0.638	(0.72–3.43)

*HM* Herbal medicine

The nonusers’ reasons for not using herbal medicines are shown in Table 6. The highest percentage explained that they feel they are healthy and have no need for herbal medicines or they do not have enough information about herbal medicines. There was a significant association between the nonuse of herbal medicines and the mentioned reasons (*p* < 0.05).

**Table 6:** The reason for not using herbal products and association with Herbal usage (*n* = 172)

Reason for not using Herbals	Total	Used Herbal	Not used Herbal	Sig
I do not believe in the efficacy of the herbal product	24 (6.3)	11 (47.8)	13 (52.2)	> 0.05
I am healthy and no need for its use	67 (17.7)	46 (68.7)	21 (31.3)	> 0.05
I don’t know enough information available about herbal products	64 (16.9)	45 (70.3)	19 (29.7)	> 0.05
herbal products are expensive for me to use	7 (1.9)	6 (85.7)	1 (14.3)	> 0.05
Others	11 (2.9)	9 (81.8)	2 (18.2)	> 0.05

## Discussion

The prevalence of herbal use in this study (80.2%) was the highest when compared with findings presented in other studies from Middle Eastern areas [5, 17, 18] and the United States [19]. The majority of previous studies reported a higher rate of use of herbal medicines among hypertensive patients [20–22]. In this study, the use of herbal medicines was not associated with any of the recorded demographic factors but age. Moreover, the only associated health-related characteristic was the patient’s disease state, including specifically hypertension. On the contrary, other studies showed an association with some demographic variables such as educational level or marital status as reported by Ibrahim et al. [17]. Another survey in Turkey showed a significant association with almost all demographic variables considered [21].

Our study’s findings were consistent with those of other studies, which reported a degree of independence between sociodemographic factors and the use of herbal medicines [23]. Any discrepancy might be attributed to different perspectives and definitions of herbal medicines among different populations due to variations in the recognition and valuation of herbal medicines as well as attitudes towards herbal medicines among different cultures.

An assortment of herbal medicines is known to be applicable in managing high blood pressure, which is supported by the findings of this study and other studies conducted in developing countries [21, 24]. The low cost and acceptability of traditional herbal medicines in different cultures made users confident with adopting these products for both therapeutic and prevention reasons. Moreover, the use of herbal medicines has a historical context and is well-accepted in Islamic culture, further strengthening users’ acceptance of these products.

Reasons for not using herbal medicines are different as reported by nonusers, and no significant single reason for non usage was stated. However, the highest percentage of nonusers reported they did not believe in the efficacy of herbal medicines. Other important reasons were that the individuals felt healthy and had no need for its use and there was unavailability of adequate information about the herbal medicines. These findings might prompt manufacturers of these herbal products to disseminate more information and perform more outreach and education regarding their products.

The highest adopted products were herbal remedies, as about of half of our sample used these products, followed to a lesser extent by vitamins and minerals, and the total percentage represents less than one-quarter of our population. Our results indicated that older patients were the most frequent users of herbs, vitamins, and minerals. This can be explained by the fact that the older population has more ailments and health issues as compared with their younger counterparts and hence are likely looking for additional health and wellness support.

The reasons for the use of herbs as reported by the study population were mainly to treat diseases and to maintain health followed by preventing illness, which are logical findings in relation to the use of such herbal products. The use of herbal medicines was recommended by family and friends to the greatest extent and secondly by pharmacists, while physician recommendations were the most infrequent recommendations received. Consistently, other studies found nearly the same pattern where seekers do not ask medical advice and instead depend upon friends and family members for guidance [25, 26].

The most common side effects as reported by patients in this study were nausea and vomiting and, to a lesser extent, skin rash, which is inconsistent with the findings of other studies that found other multiple side effects including mainly skin rash as the primary unwanted effect of traditional therapy [25, 27]. Side effects and drug interactions are common among users of these herbal products, as they are users of other medications such as antihypertensive drugs; hence, health care professionals should be vigilant and educate patients regarding these issues. In addition, the lack of accurate or regulated dosing of these products is another major concern. All of these aspects represent potential sources of debate among health professionals about the risk–benefit ratio and effectiveness of these products.

### Limitations

Study participant recruitment was done inside universities, so most of the study sample was from specific age groups spanning students’ ages. Another limitation was the convenience sampling method used in this study. Our findings may not be extrapolated to the broader population of Jordan or to those of other countries.

## Conclusion

We found that the use of herbal medicines is common among the study population, including specifically hypertensive patients, in Jordan, and the same is true among other Middle East populations. Demographic characteristics are not significantly related to the use of herbal medicines. The only determinant of the use of these products is the presence of elevated blood pressure. Nausea and vomiting were the most common side effects reported by consumers of herbal medicines. It is worth knowing that herbal products are not risk-free and the risk of drug interactions is not currently well-studied, so further research in this area is warranted and health care professionals should suggest caution to patients where appropriate.

## Data Availability

The data that support the findings of this study are available from the corresponding author upon reasonable request.
